# Pulsed Radiofrequency as a Promising Strategy for Persistent Morton’s Neuroma Pain

**DOI:** 10.7759/cureus.91723

**Published:** 2025-09-06

**Authors:** Yassine Benghali, Nkhili Yassine, Abderrahim Lachhab, Ahmed Amine El Oumri

**Affiliations:** 1 Physical Medicine and Rehabilitation, Faculty of Medicine Mohammed First University of Oujda/University Hospital Center Mohammed VI, Oujda, MAR; 2 Physical Medicine and Rehabilitation, Faculty of Medicine and Pharmacy, Mohammed First University of Oujda, Oujda, MAR; 3 Faculty of Medicine, Mohammed First University, Oujda, MAR; 4 Physical Medicine and Rehabilitation, Mohammed VI University Hospital, Oujda, MAR; 5 Physical Medicine and Rehabilitation, Faculty of Medicine of Oujda/Mohammed VI University Hospital of Oujda/Mohammed First University of Oujda, Oujda, MAR

**Keywords:** chronic pain management, foot and ankle outcome score(faos), forefoot pain, minimally invasive treatment, morton's neuroma, neuromodulation therapies, pulsed radiofrequency treatment, ultrasound-guided

## Abstract

Morton’s neuroma is a prevalent cause of forefoot pain, often proving refractory to conventional conservative treatments. This case report describes a 25-year-old female nurse presenting with debilitating Morton’s neuroma located in the second intermetatarsal space, who had not responded to prior conservative therapies. The patient underwent ultrasound-guided pulsed radiofrequency (PRF) treatment. Following the procedure, she experienced significant pain reduction, with her visual analog scale (VAS) score improving from 9/10 to 1/10. This case underscores the clinical efficacy of PRF as a safe, minimally invasive, and effective therapeutic option for patients suffering from refractory Morton’s neuroma, suggesting its valuable role within the comprehensive management algorithm for this condition.

## Introduction

Morton’s neuroma, a common cause of forefoot pain, is characterized by perineural fibrosis and degeneration of the common digital nerve, most frequently affecting the third intermetatarsal space [1]. Patients typically present with a burning sensation, electric shock-like pain, and numbness, often exacerbated by tight footwear and prolonged standing, as observed in the present case [2, 3]. Diagnosis is primarily clinical, supported by a positive Mulder’s sign, and confirmed through imaging modalities such as ultrasound, which reveals a hypoechoic intermetatarsal mass [3].

In more recent decades, pulsed radiofrequency (PRF), as proposed by Deniz et al. in 2015, has been adopted as an alternative method in the treatment of several painful disorders [4].

While conservative management, including infiltrative therapies like corticosteroid injections, is generally the first-line treatment, its effectiveness is variable; symptoms may persist despite these measures [5, 6]. Corticosteroid injections, as a form of infiltrative treatment, have shown mixed results, with some systematic reviews suggesting temporary relief but limited long-term efficacy [5, 7]. When conservative approaches fail, various interventional treatments are considered, ranging from infiltrative therapies to surgical excision [6]. Surgical neurectomy, though definitive, carries risks such as stump neuroma formation and persistent numbness [6].

PRF modulates nerve function without the destructive thermal effects associated with conventional radiofrequency, acting through distinct cellular and molecular mechanisms [8, 9]. This case report aims to highlight the effectiveness of PRF in providing significant pain relief and functional improvement in a patient with Morton’s neuroma refractory to conservative management.

## Case presentation

A 25-year-old female nurse, with no significant medical history, presented with a three-month history of progressive forefoot pain localized between the second and third toes. The pain was described as a burning sensation and electric shock-like pain, characteristic of neuropathic involvement. Despite initial use of anti-inflammatory and analgesic medications, symptoms failed to improve. Her initial DN4 score was 5/10, indicating the presence of neuropathic pain. This score was derived from the patient's reported symptoms of burning and electric shock-like pain, combined with clinical findings consistent with sensory abnormalities, including hypoesthesia to touch, hypoesthesia to pinprick, and mechanical allodynia. The pain intensity was initially assessed at a VAS score of 9/10, indicating severe pain. Her occupation, involving prolonged standing and wearing tight shoes, exacerbated the symptoms. On clinical examination, no biomechanical alterations were noted in the knee or ankle, further localizing the pain to the forefoot. A biological workup, including normal leukocyte count, C-reactive protein (CRP), erythrocyte sedimentation rate (ESR), rheumatoid factor (RF), and anti-CCP antibodies, was performed and found to be normal, with these results detailed in Table 1.

**Table 1:** Initial Laboratory Workup and Normal Values CCP: Cyclic Citrullinated Peptide

Analysis	Results	Normal Values
Leukocytes	7,200 cells/µL	4,000–11,000 cells/µL
C-Reactive Protein (CRP)	3 mg/L	< 5 mg/L
Erythrocyte Sedimentation Rate (ESR)	12 mm/h	Men: < 15 mm/h
		Women: < 20 mm/h
Rheumatoid Factor (RF)	12 IU/mL	< 20 IU/mL
Anti-CCP Antibodies	2 U/mL	< 5 U/mL

**Figure 1:**
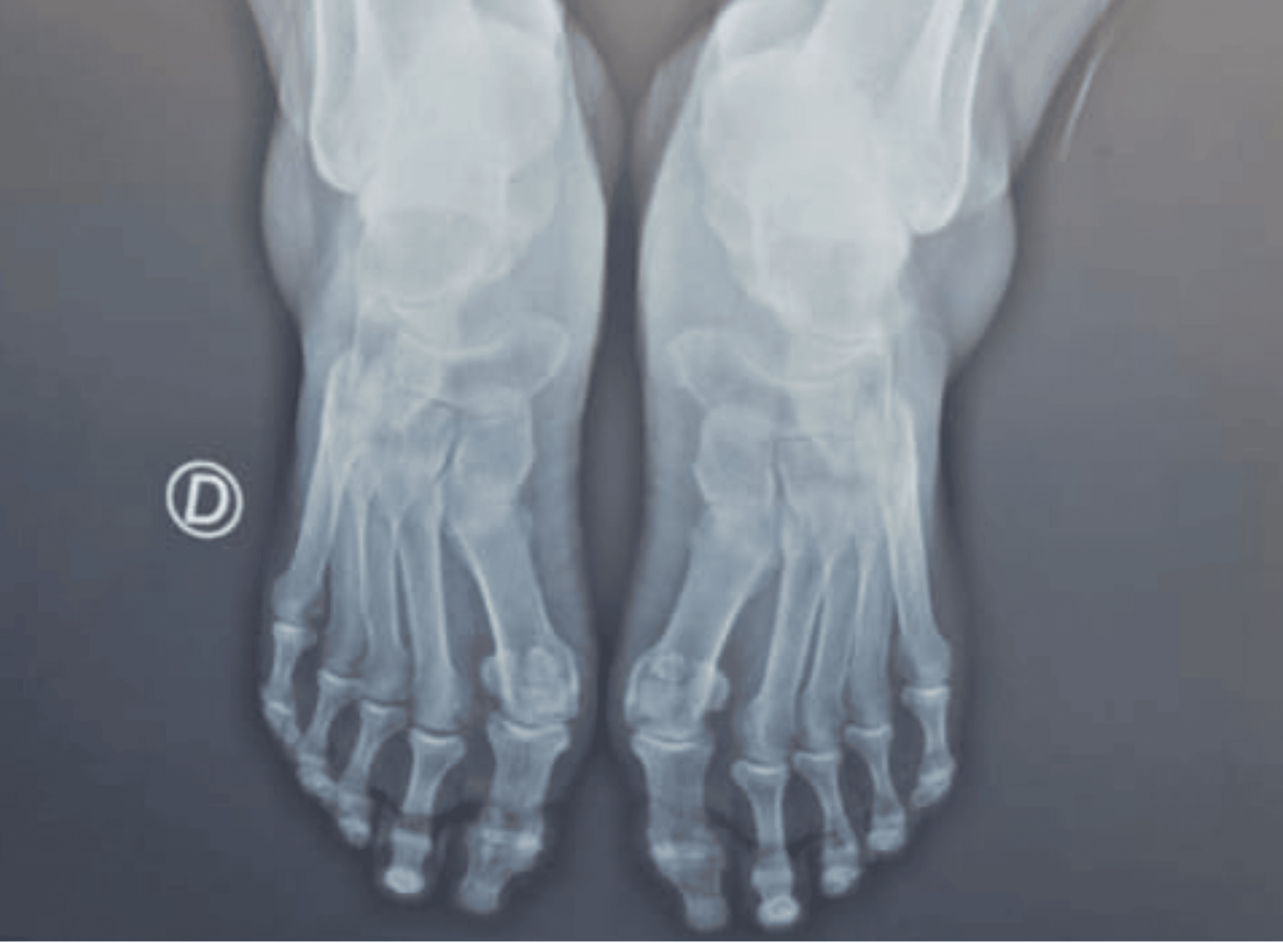
Weight-bearing dorsoplantar X-ray of the forefoot D: right foot

**Figure 2:**
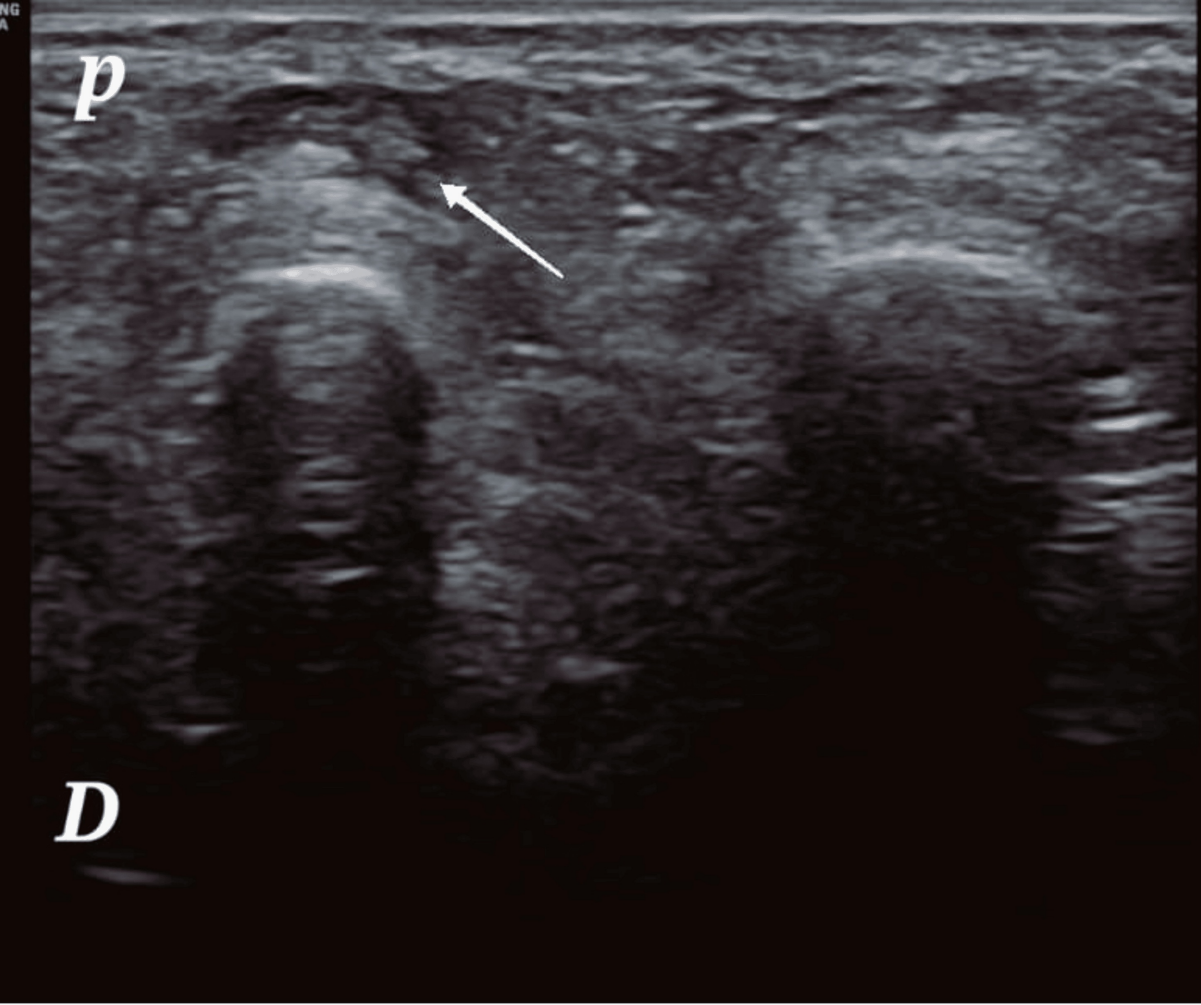
Ultrasound image of a Morton’s neuroma. p – Indicates the plantar aspect of the foot. D – Indicates the dorsal aspect of the foot.

The patient underwent a single PRF session under ultrasound guidance. Weight-bearing dorsoplantar X-rays of the forefoot were normal (Figure 1), ruling out an obvious bony etiology for the pain. An ultrasound of the forefoot was subsequently performed, which confirmed the diagnosis by revealing a hypoechoic, ovoid lesion, approximately 6 mm in diameter, located in the second intermetatarsal space. The lesion showed no perceptible Doppler signal, which is consistent with the characteristics of a Morton’s neuroma (Figure 2).

**Figure 3:**
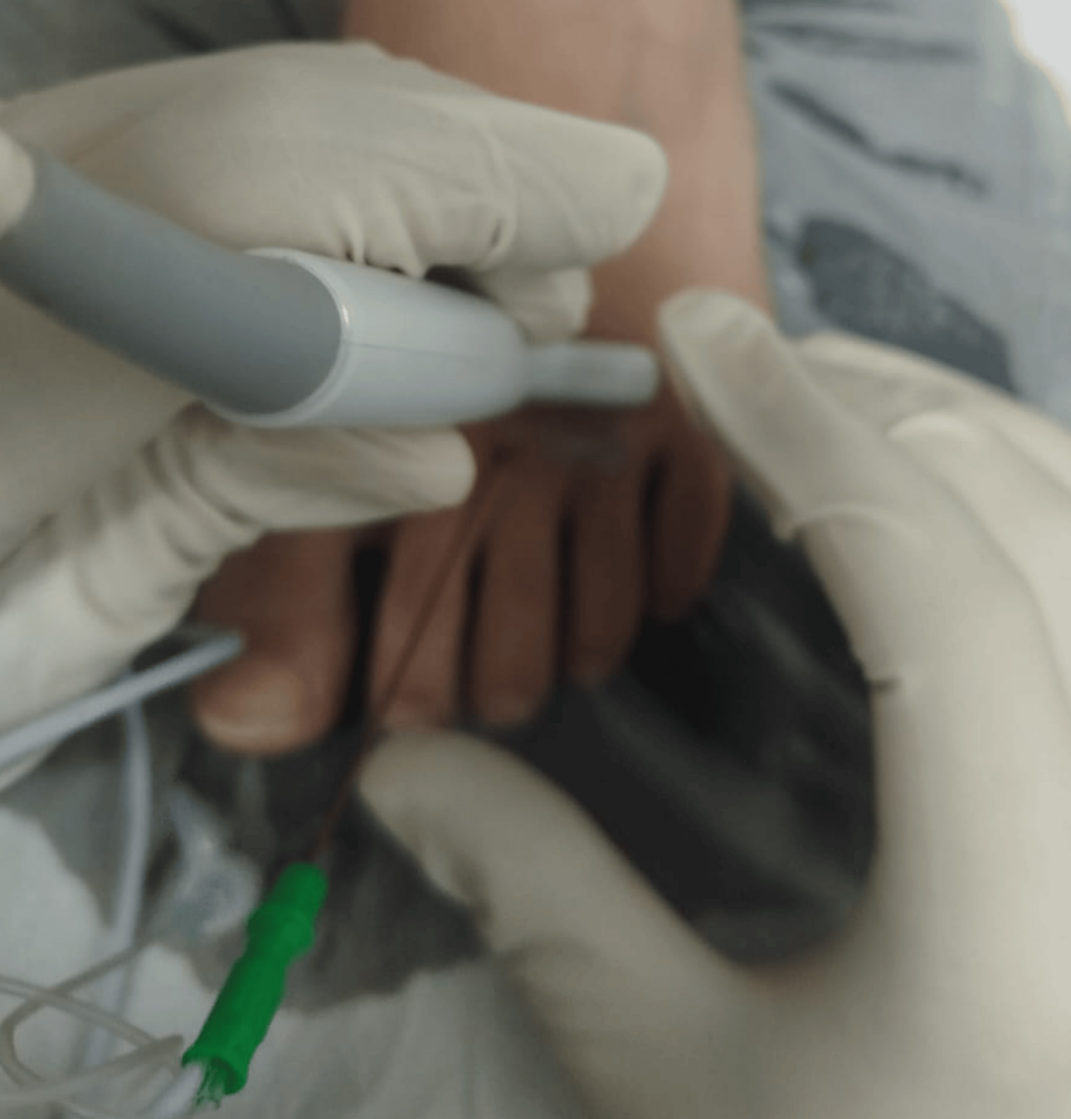
Ultrasound-guided pulsed radiofrequency procedure (Out-of-Plane Approach)

PRF was selected for its ability to modulate nerve signals without inducing thermal damage. The procedure was performed using a TOP LESION generator TLG 20 radiofrequency device. The patient was positioned in decubitus dorsal (supine) with the affected knee flexed at 60 degrees and the foot in approximately 20 degrees of plantar flexion. Under ultrasound guidance, a 22-gauge needle was precisely advanced into the second intermetatarsal space using an out-of-plane approach to target the neuroma. Figure 3 illustrates this procedure. PRF energy was then delivered at a temperature of 42°C for 8 minutes. No transient increase in pain was observed immediately after the procedure, which is consistent with the non-destructive nature of PRF.

**Figure 4:**
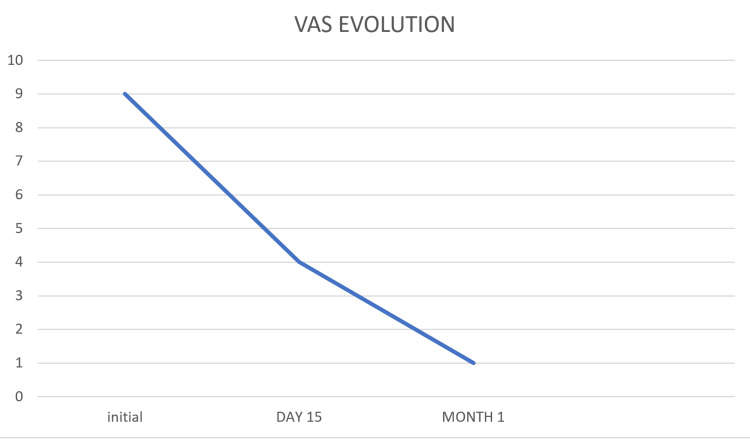
Visual analog scale (VAS) Score reduction post-procedure

Post-procedure, the patient experienced significant pain relief. The visual analog scale (VAS) score dropped from a baseline of 9/10 to 4/10 at day 15, and further decreased to 1/10 at one month. Figure 4 graphically represents this diminution in VAS scores. This highlights the effectiveness of PRF in achieving substantial symptom relief.

## Discussion

Morton’s neuroma, though defined in the introduction, represents a persistent clinical challenge due to the often debilitating nature of the forefoot pain it causes [1, 2]. Classically, it is most frequently located in the third intermetatarsal space (between the 3rd and 4th metatarsals), but also commonly in the second intermetatarsal space (between the 2nd and 3rd metatarsals) [1]. In our patient, the neuroma was specifically located between the second and third metatarsals.

The diagnostic process primarily relies on clinical evaluation, notably the identification of Mulder's sign [2, 3]. Imaging plays an essential complementary role in confirming clinical suspicion and excluding other forefoot pathologies [1, 3]. Ultrasound is particularly favored for its ability to directly visualize the characteristic hypoechoic lesion of the neuroma - a hypoechoic mass with no Doppler signal - and for guiding interventional procedures [1, 3]. Furthermore, a general biological workup (including leukocyte count, CRP, ESR, RF, and anti-cyclic citrullinated peptide (anti-CCP) antibodies) is also performed for differential diagnosis to rule out inflammatory or systemic conditions. The normal results of these analyses in our patient allowed us to exclude a systemic etiology for her pain.

Regarding management, the therapeutic progression generally begins with conservative measures, such as footwear modifications and the use of analgesics [6]. Nevertheless, when these initial approaches prove insufficient, as was the case for the presented patient, interventional options become necessary. Among these, infiltrative therapies, including corticosteroid injections or sclerosing agents like alcohol, are commonly employed [5, 7]. However, the efficacy of corticosteroid injections can be limited in duration, and variability in long-term results is reported in the literature [5, 7]. As a last resort, surgical excision of the neuroma is a definitive solution, although it may lead to complications such as stump neuroma formation or persistent dysesthesias [6].

In this context, PRF has emerged as a promising, minimally invasive therapeutic modality. PRF stands out due to its unique mechanism of action: it modulates nerve conduction without causing significant thermal tissue destruction, unlike ablative techniques [8, 9]. This process of cellular and molecular neuromodulation allows for pain relief while preserving nerve integrity, thereby minimizing the risk of irreversible sequelae [8, 9]. The application of PRF is optimally performed under ultrasound guidance, which ensures enhanced precision in targeting the affected digital nerve and minimizes risks to adjacent structures [4]. The efficacy of PRF in improving pain associated with Morton’s neuroma is increasingly documented, and the results observed in our patient corroborate these data. Studies have demonstrated that ultrasound-guided PRF can lead to a significant reduction in pain scores and functional improvement in patients [4]. A recent systematic review and meta-analysis of radiofrequency techniques for Morton’s neuroma have also highlighted positive results, affirming the potential of this approach [10]. The improvement in symptoms observed in our case presentation, particularly the notable pain reduction on the VAS after the failure of more conventional treatments, illustrates the relevance of PRF as a viable therapeutic option. Its safety profile and minimally invasive nature make it an attractive alternative for patients whose symptoms persist.

## Conclusions

Morton’s neuroma represents a common and often debilitating cause of forefoot pain, frequently proving refractory to conservative management. This case report underscores the clinical efficacy of PRF in achieving significant pain relief and functional improvement for a patient with Morton’s neuroma unresponsive to conventional treatments. PRF, with its distinct non-destructive neuromodulatory mechanism and precision afforded by ultrasound guidance, offers a valuable, minimally invasive alternative in the therapeutic algorithm. The favorable outcome observed in this case supports PRF's role as an effective and safe intervention for persistent Morton’s neuroma symptoms, warranting further investigation into its broader application and long-term benefits.
